# Prevalence and associated factors of cross-sectional and incident self-reported arthritis or rheumatism among a national community sample of middle-aged and older adults in Thailand

**DOI:** 10.3389/fpubh.2023.1064751

**Published:** 2023-02-01

**Authors:** Supa Pengpid, Karl Peltzer

**Affiliations:** ^1^Department of Health Education and Behavioral Sciences, Faculty of Public Health, Mahidol University, Bangkok, Thailand; ^2^Department of Public Health, Sefako Makgatho Health Sciences University, Pretoria, South Africa; ^3^Department of Psychology, University of the Free State, Bloemfontein, South Africa; ^4^Department of Psychology, College of Medical and Health Sciences, Asia University, Taichung, Taiwan

**Keywords:** arthritis (MeSH), aging, rheumatoid arthritis, survey, Thailand

## Abstract

**Background:**

The study aimed to assess the prevalence and associated factors of cross-sectional and incident arthritis or rheumatism among a national community sample of middle-aged and older adults in Thailand.

**Methods:**

We analyzed cross-sectional and longitudinal data from two consecutive waves (2015 and 2017) of the Health, Aging, and Retirement in Thailand (HART) study. Arthritis or rheumatism (SRA) was assessed by self-reported health care provider diagnosis.

**Results:**

The cross-sectional (baseline) sample included 5,616 participants (≥45 years, median age 66 years, interquartile range 57 to 76 years) and the incident (follow-up) sample included 3,545 participants. The prevalence of SRA in the cross-sectional sample (baseline) was 4.0% and in the incident (follow-up) sample 5.3%. In the cross-sectional multivariable model, obesity class I (aOR: 1.78, 95% CI: 1.19 to 2.67), obesity class II (aOR: 1.82, 95% CI: 1.02 to 3.25), hypertension (aOR: 1.90, 95% CI: 1.35 to 2.66), brain disease and/or psychiatric problems (aOR: 4.79, 95% CI: 2.27 to 10.62), sleep problem (aOR: 1.45, 95% CI: 1.01 to 2.07) and prescription drug use (aOR: 1.63, 95% CI: 1.14 to 2.33) were positively associated, and not in the labor force (aOR: 0.53, 95% CI: 0.34 to 0.84), and employed (aOR: 0.63, 95% CI: 0.41 to 0.99) were negatively associated with SRA. In the incident multivariable model, obesity class I (aOR: 1.78, 95% CI: 1.17 to 3.61), obesity class II (aOR: 2.01, 95% CI: 1.12 to 3.61), poor mental health (aOR: 1.69, 95% CI: 1.19 to 2.41), and functional disability (aOR: 2.04, 95% CI: 1.01 to 4.13) were positively associated, and current alcohol use (aOR: 0.50, 95% CI: 0.25 to 0.99) was negatively associated with SRA.

**Conclusion:**

The middle and older Thai adults had a low prevalence and incidence of SRA, and several physical and mental risk factors for cross-sectional and/or incident SRA were identified.

## Introduction

A significant global burden of disability can be attributed to arthritis (1, 2). Worldwide, in the adult population, the prevalence of knee osteoarthritis was 3.8% (16.0% in persons ≥40 years), hip osteoarthritis 0.85% and rheumatoid arthritis was 0.24% (1–3). Among predominantly older adults in six low-and middle-income countries, the prevalence of self-reported arthritis (SRA) was 19.9% among women and 14.1% among men (4). Among older adults (≥50 years) in South Africa, 24.7% had SRA (5), in India (≥50 years) 14.7% (6), and in China (≥45 years) 31.4% had SRA (7). We were unable to identify national data on the prevalence, incidence, and correlates of SRA among the aging population in Thailand, which led to the study.

According to previous studies (7–22), factors associated with arthritis include sociodemographic factors, health risk behaviors, poor mental health, and chronic conditions. Sociodemographic factors associated with arthritis include older age (7–11), female sex (7–11), higher economic status (9) and lower education (10, 12). Specific health risk behaviors, such as smoking (13), low physical activity or sedentary behavior (12, 14–16), non-alcohol use (17) and obesity (5, 8, 14, 18) have shown to increase the risk of arthritis. Moreover, poor mental health (5, 19, 20), including sleep problems (19) and depressive symptoms (10, 18, 21), increased the odds of arthritis. Certain chronic conditions, such as hypertension (7, 10), cardiovascular disease (7, 22), kidney and chronic lung disease (7), and functional disability (10, 11, 19) were also found to be associated with arthritis. Factors associated with incident arthritis include increasing age (7, 23), female sex (7, 23), physical activity (7), physical inactivity (23) cardiovascular disease (7), obesity (24, 25), lower well-being (26), sleep problems (27) and depression (23, 28).

The study aimed to assess for the first time the prevalence, incidence, and factors associated with SRA among middle-aged and older adults in a national community-based sample in Thailand in 2015 and 2017.

## Methods

### Sample and procedure

We analyzed cross-sectional and longitudinal data from two consecutive waves in 2015 and 2017 of the Health, Aging, and Retirement in Thailand (HART) study. From the total population and household data from the Department of Provincial Administration (DOPA), Ministry of Interior, in Thailand, a three-stage stratified random sampling was used. In stage 1, in each of 6 regions (Bangkok & Vicinity, East, North, Northeast, and South) in Thailand, one small province (< 250,000 people ≥45 years) and one large province (>250,000 people ≥45 years) was selected, except for the East where only one province was selected. In stage 2, each province is classified into urban areas (number of blocks) and rural areas (number of villages). In stage 3, 5,600 households were selected from the sampled blocks and villages. In each household, one person (≥45 years) was randomly selected, which was the inclusion criterium. Proxy interviews were conducted for frail participants (29). The 2015 survey (from February to July) (*N* = 5,616), and the 2017 survey (from January to June 2017) included 3,708 members of the 2015 HART cohort (192 died during follow-up or 4.3% of the baseline respondents who were in the study area; 1,554 moved away from the study area; 270 declined participation and the response rate: 72.33% and the retention rate: 66.03%). Attrition analysis found that those who were not followed-up were more likely more educated, Buddhist and male, while there were no significant differences in terms of other sociodemographic and all health variables. Participants were interviewed at their homes by trained field workers after written informed consent was obtained. The study was approved by the “Ethics Committee in Human Research, National Institute of Development Administration – ECNIDA (ECNIDA 2020/00012).”

### Measures

*Arthritis or rheumatism* was assessed with self-reported health care provider diagnosis. Self-report is a reliable method of identifying arthritis or rheumatism in large population-based surveys (7).

*Other chronic conditions* were evaluated by self-reported health care provider diagnosed conditions, including hypertension, diabetes, lung diseases, emphysema, cardiovascular diseases, heart disease, heart failure, kidney diseases, liver diseases, emotional/nervous or psychiatric disease, brain diseases and Alzheimer's disease.

*Sociodemographic information* included age, sex, educational level, religion, and annual income quartile (30).

*Employment status*. First, participants were asked if they had a job (working for an employed, self-employed, or working for family or relative's business) now (Yes/No). Participants who had no job currently were asked if they worked before but retired, worked before and intended to work in the future but were currently not looking for a job, or never had a job. Retired individuals were defined as having no job at the moment and not intending to work in the future, those without a job and intending to work in the future as unemployed, and those who never had a job as ‘not in the workforce' (31).

*Tobacco smoking* was sourced from the item, “Have you ever smoked cigarettes?” (response options: “1 = yes, and still smoke now, 2 = yes, but quit smoking, and 3 = never”).

*Alcohol use* was sourced from the item, “Have you ever drunk alcoholic beverages such as liquor, beer or wine?” (response options: 1 = yes, and still drinking now, 2 = yes, but do not drink now, and 3 = never).

*Physical activity* in the past week was classified as “none = inactivity, 1–149 min/week = low activity, and ≥150 min/week = high activity” (32).

*Body Mass Index* (BMI) was assessed by self-reported body weight and height, and classified into “underweight (< 18.5 kg/m^2^), normal weight (18.5–22.9 kg/m^2^), overweight (23–24.9 kg/m^2^), obesity class I (25–29.9 kg/m^2^), and obesity class II (30 kg/m^2^)” (33).

*Prescription drug use* was assessed with the question, “In the last 2 years, did you use any prescription drug?” (Yes/No).

*Functional disability* was defined as any of four activity of daily living (ADL) limitations (34), previously found a valid measure in older adults in Thailand (35) (Cronbach's α = 0.94 at wave 1).

*Probable depression* (≥10 scores) was assessed using the Center for Epidemiologic Studies Depression (CES-D-10) scale (36), previously found a valid measure in Thailand (37, 38).

Factors associated with incident arthritis include increasing age [7/23], female sex (7, 23), physical activity (7), physical inactivity (23) cardiovascular disease (7), obesity (24, 25), lower well-being (26), sleep problems (27) and depression (23, 28). The CES-D10 had a reliability coefficient of 0.78.

*Mental health* s*tatus* was assessed with the question: “In general, how would you rate your mental health status?” Responses ranged from 0 = very poor to 100 excellent, and poor mental health was defined as 0 to < 80 and good mental health as 80–100.

*Sleep problem* was defined as almost always or often (vs. sometimes or very rarely or never) having trouble falling asleep/insomnia in the past week.

### Data analysis

The proportion of older adults with cross-sectional and incident SRA are presented with frequencies and percent. Pearson Chi-square tests are used to compare characteristics among groups. The first logistic regression model estimated odds ratios (OR) and confidence intervals (CI) for cross-sectional SRA, and the second model compared baseline without SRA with incident SRA. Variables significant in univariable analysis were included in the multivariable models. *p* ≤ 0.05 was considered statistically significant. All statistical analyzes were performed with StataSE 15.0 (College Station, TX, USA).

## Results

### Sample characteristics

The cross-sectional (baseline) sample in 2015 included 5,616 participants (≥45 years, median age 66 years, interquartile range 57 to 76 years) and the incident (follow-up) sample in 2017 included 3,545 participants. The prevalence of SRA in the cross-sectional sample (baseline) was 4.0% and in the incident (follow-up) sample 5.3%. The binary analysis in the cross-sectional sample found that age, religion, employment status, smoking status, body mass index, probable depression, mental health, sleep problem, hypertension, diabetes, cardiovascular disease, kidney disease, brain disease or psychiatric problems, and prescription drug use differed significantly between people with SRA and without SRA. Binary analysis in the incident sample found that age, employment status, alcohol use, body mass index, probable depression, mental health status, sleep problem, hypertension, cardiovascular disease, kidney disease, prescription drug use and functional disability differed significantly between people with SRA and without SRA (see Table 1).

**Table 1:** Cross-sectional and incident arthritis or rheumatism.

**Variables**	**Subcategory**	**Cross-sectional arthritis or rheumatism**		***p*-value**	**Incident arthritis or rheumatism**		***p*-value**

		**No**	**Yes**		**No**	**Yes**	
All		5,392 (96.0)	224 (4.0)		3,356 (94.7)	189 (5.3)	
Age (in years)	45–54	1,078 (97.6)	27 (2.4)	0.003	635 (96.2)	25 (3.8)	0.018
	55–64	1,449 (96.6)	51 (3.4)		927 (95.8)	41 (4.2)	
	65–74	1,305 (95.3)	65 (4.7)		848 (93.8)	56 (6.2)	
	75 or more	1,560 (95.1)	81 (4.9)		946 (93.4)	67 (6.6)	
Sex	Female	2,809 (95.9)	121 (4.1)	0.573	1782 (94.2)	109 (5.8)	0.220
	Male	2,583 (96.2)	103 (3.8)		1,574 (95.2)	80 (4.8)	
Education	≤ Elementary	4,389 (95.8)	191 (4.2)	0.175	2,810 (94.5)	165 (5.5)	0.217
	>Elementary	983 (96.8)	33 (3.2)		538 (95.7)	24 (4.3)	
Religion	Muslim or other	337 (94.0)	24 (6.0)	0.035	267 (93.0)	20 (7.0)	0.199
	Buddhist	5,008 (96.2)	200 (3.8)		3,087 (94.8)	169 (5.2)	
Employment status	Retired	1314 (94.1)	83 (5.9)	< 0.001	821 (93.5)	57 (6.5)	0.002
	Unemployed	324 (95.3)	16 (4.7)		214 (90.7)	22 (9.3)	
	Not in labor force	1,089 (96.3)	42 (3.7)		679 (95.0)	36 (5.0)	
	Employed	2,554 (97.0)	78 (3.0)		1,598 (95.9)	69 (4.1)	
Income quartile	Low	1,336 (95.2)	67 (4.8)	0.174	813 (94.8)	45 (5.2)	0.254
	Lower middle	1,320 (95.7)	59 (4.3)		834 (94.1)	52 (5.9)	
	Upper middle	1,367 (96.3)	52 (3.7)		869 (93.9)	56 (6.1)	
	High	1,369 (96.7)	46 (3.3)		840 (95.9)	36 (4.1)	
Alcohol use	Never	4,350 (96.0)	180 (4.0)	0.069	2,699 (94.2)	167 (5.8)	0.010
	Past	368 (94.1)	23 (5.9)		237 (95.2)	12 (4.8)	
	Current	674 (97.0)	21 (3.0)		420 (97.7)	10 (2.3)	
Smoking tobacco	Never	4,310 (96.1)	173 (3.9)	0.013	2,678 (94.4)	160 (5.6)	0.266
	Past	398 (93.4)	28 (6.6)		262 (96.0)	11 (4.0)	
	Current	684 (96.7)	23 (3.3)		416 (95.9)	18 (4.1)	
Physical activity	None	3,251 (96.3)	125 (3.7)	0.180	1,953 (94.1)	123 (5.9)	0.137
	1–149 minutes/week	1,297 (95.2)	66 (4.8)		849 (95.2)	43 (4.8)	
	≥150 minutes/week	844 (96.2)	33 (3.8)		554 (96.0)	23 (4.0)	
Body mass index	Normal	1,845 (96.5)	67 (3.5)	0.028	1l151 (95.8)	51 (4.2)	0.011
	Under	542 (97.0)	17 (3.0)		325 (93.9)	21 (6.1)	
	Overweight	968 (96.1)	39 (3.9)		603 (95.4)	29 (4.6)	
	Obesity I	1l164 (94.9)	63 (5.1)		714 (92.8)	55 (7.2)	
	Obesity II	330 (94.0)	21 (6.0)		200 (91.7)	18 (8.3)	
Probable depression	No	4,367 (96.4)	161 (3.6)	0.018	,2727 (95.0)	144 (5.0)	0.029
	Yes	605 (94.5)	35 (5.5)		362 (92.3)	30 (7.7)	
Mental health	Good (80–100)	3,705 (96.5)	135 (3.5)	0.004	2,367 (95.6)	108 (4.4)	< 0.001
	Poor (< 80)	1,562 (94.8)	85 (5.2)		917 (92.4)	75 (7.6)	
Sleep problem	Rarely/sometimes	4,470 (96.5)	164 (3.5)	< 0.001	2,809 (95.2)	141 (4.8)	0.007
	Often/Mostly	859 (93.8)	57 (6.2)		516 (92.5)	42 (7.5)	
Hypertension	No	3,565 (97.3)	100 (2.7)	< 0.001	2,210 (95.5)	105 (4.5)	0.004
	Yes	1,827 (93.6)	124 (6.4)		1,146 (93.2)	84 (6.8)	
Diabetes	No	4,591 (96.3)	176 (3.7)	0.007	2,866 (94.9)	153 (5.1)	0.094
	Yes	801 (94.3)	48 (5.7)		490 (93.2)	36 (6.8)	
Cardiovascular disease	No	5,136 (96.2)	203 (3.8)	0.002	3,192 (94.9)	172 (5.1)	0.013
	Yes	256 (92.4)	21 (7.6)		164 (90.6)	17 (9.4)	
Kidney diseases	No	5,301 (96.2)	210 (3.8)	< 0.001	33,00 (94.8)	182 (5.2)	0.039
	Yes	91 (86.7)	14 (13.3)		56 (88.9)	7 (11.1)	
Brain diseases/ psychiatric problems	No	5,340 (96.2)	211 (3.8)	< 0.001	3,325 (94.7)	185 (5.3)	0.107
	Yes	52 (80.0)	13 (20.0)		31 (88.6)	4 (11.4)	
Lung diseases	No	5,347 (96.0)	220 (4.0)	0.134	3,325 (94.7)	186 (5.3)	0.362
	Yes	45 (91.8)	4 (8.2)		31 (91.2)	3 (8.8)	
Prescription drug	No	4,484 (96.5)	162 (3.5)	< 0.001	2,785 (95.5)	144 (4.9)	0.016
	Yes	908 (93.6)	62 (6.4)		571 (92.7)	45 (7.3)	
Functional disability	No	5,110 (96.2)	203 (3.8)	0.058	3,212 (95.0)	169 (5.0)	< 0.001
	Yes	188 (93.5)	13 (6.5)		516 (92.5)	13 (11.9)	

### Cross-sectional associations with SRA

In the multivariable model, brain disease and/or psychiatric problems (aOR: 4.79, 95% CI: 2.27 to 10.62), hypertension (aOR: 1.90, 95% CI: 1.35 to 2.66), obesity class II (aOR: 1.82, 95% CI: 1.02 to 3.25), obesity class I (aOR: 1.78, 95% CI: 1.19 to 2.67), prescription drug use (aOR: 1.63, 95% CI: 1.14 to 2.33), and sleep problem (aOR: 1.45, 95% CI: 1.01 to 2.07) were positively associated, and not in the labor force (aOR: 0.53, 95% CI: 0.34 to 0.84), and employed (aOR: 0.63, 95% CI: 0.41 to 0.99) were negatively associated with SRA (see Table 2).

**Table 2:** Cross-sectional associations with arthritis, Health, Aging, and Retirement in Thailand (HART).

**Variables**	**Subcategory**	**COR (95% CI)**		**AOR (95% CI)**	
Age (in years)	Scale	1 (Reference)	< 0.001	1 (Reference)	0.090
		1.02 (1.01 to 1.03)		1.01 (1.00 to 1.03)	
Sex	Female	1 (Reference)	0.573	—	
	Male	0.93 (0.71 to 1.21)			
Education	≤ Elementary	1 (Reference)	0.176	—	
	>Elementary	0.77 (0.53 to 1.12)			
Religion	Muslim or other	1 (Reference)	0.036	1 (Reference)	0.082
	Buddhist	0.63 (0.41 to 0.97)		0.63 (0.37 to 1.06)	
Employment status	Retired	1 (Reference)	0.379	1 (Reference)	0.685
	Unemployed	0.78 (0.45 to 1.35)	0.010	0.85 (0.46 to 1.60)	0.007
	Not in labor force	0.61 (0.41 to 0.89)	< 0.001	0.53 (0.34 to 0.84)	0.044
	Employed	0.48 (0.35 to 0.66)		0.63 (0.41 to 0.99)	
Income quartile	Low	1 (Reference)	0.509	1 (Reference)	0.597
	Lower middle	0.89 (0.62 to 1.28)	0.143	0.89 (0.59 to 1.36)	0.575
	Upper middle	0.76 (0.52 to 1.10)	0.040	0.87 (0.55 to 1.40)	0.720
	High	0.67 (0.46 to 0.98)		0.91 (0.54 to 1.54)	
Alcohol use	Never	1 (Reference)	0.070	—	
	Past	1.51 (0.97 to 2.36)	0.226		
	Current	0.75 (0.48 to 1.19)			
Smoking tobacco	Never	1 (Reference)	0.008	1 (Reference)	0.071
	Past	1.75 (1.16 to 2.65)	0.433	1.57 (0.96 to 2.55)	0.713
	Current	0.84 (0.54 to 1.30)		1.10 (0.66 to 1.86)	
Physical activity	None	1 (Reference)	0.072	—	
	1–149 min/week	1.32 (0.98 to 1.80)	0.933		
	≥150 min/week	1.02 (0.69 to 1.50)			
Body mass index	Normal	1 (Reference)	0.864	1 (Reference)	0.713
	Under	0.86 (0.50 to 1.48)	0.613	0.92 (0.51 to 1.65)	0.183
	Overweight	1.11 (0.74 to 1.66)	0.026	1.32 (0.84 to 2.05)	0.003
	Obesity I	1.49 (1.05 to 2.12)	0.029	1.78 (1.19 to 2.67)	0.041
	Obesity II	1.75 (1.06 to 2.90)		1.82 (1.02 to 3.25)	
Probable depression	No	1 (Reference)	0.019	1 (Reference)	0.117
	Yes	1.57 (1.08 to 2.28)		1.42 (0.92 to 2.20)	
Mental health	Good (80-100)	1 (Reference)	0.005	1 (Reference)	0.069
	Poor (< 80)	1.49 (1.13 to 1.97)		1.39 (0.98 to 1.90)	
Sleep problem	Rarely/sometimes	1 (Reference)	2.47	1 (Reference)	0.044
	Often/Mostly	1.81 (1.33 to 2.47)		1.45 (1.01 to 2.07)	
Hypertension	No	1 (Reference)	< 0.001	1 (Reference)	< 0.001
	Yes	2.40 (1.85 to 3.17)		1.90 (1.35 to 2.66)	
Diabetes	No	1 (Reference)	0.008	1 (Reference)	0.397
	Yes	1.56 (1.13 to 2.17)		0.84 (0.56 to 1.26)	
Cardiovascular disease	No	1 (Reference)	0.002	1 (Reference)	0.265
	Yes	2.08 (1.30 to 3.31)		1.37 (0.79 to 2.39)	
Kidney diseases	No	1 (Reference)	< 0.001	1 (Reference)	0.148
	Yes	3.88 (2.18 to 6.93)		1.78 (0.81 to 3.90)	
Brain diseases and psychiatric problems	No	1 (Reference)	< 0.001	1 (Reference)	< 0.001
	Yes	6.33 (3.39 to 11.80)		4.79 (2.17 to 10.62)	
Lung diseases	No	1 (Reference)	0.143	—	
	Yes	2.16 (0.77 to 6.06)			
Prescription drug	No	1 (Reference)	< 0.001	1 (Reference)	0.008
	Yes	1.89 (1.40 to 2.55)		1.63 (1.14 to 2.33)	
Functional disability	No	1 (Reference)	0.061	—	
	Yes	1.74 (0.98 to 3.11)			

COR, Crude Odds Ratio; AOR, Adjusted Odds Ratio; CI, Confidence Interval.

### Associations with incident SRA

In the multivariable model, functional disability (aOR: 2.04, 95% CI: 1.01 to 4.13), obesity class II (aOR: 2.01, 95% CI: 1.12 to 3.61), obesity class I (aOR: 1.78, 95% CI: 1.17 to 3.61), and poor mental health (aOR: 1.69, 95% CI: 1.19 to 2.41), were positively associated, and current alcohol use (aOR: 0.50, 95% CI: 0.25 to 0.99) was negatively associated with SRA (see Table 3).

**Table 3:** Longitudinal associations with incident arthritis, HART 2015–2017.

**Variables**	**Subcategory**	**COR (95% CI)**		**AOR (95% CI)**	
Age (in years)	Scale	1 (Reference)	0.653	1 (Reference)	0.573
		1.12 (0.68 to 1.87)	0.036	1.01 (0.99 to 1.02)	
		1.60 (1.04 to 2.72)	0.015		
		1.80 (1.12 to 2.88)			
Sex	Female	1 (Reference)	0.219	—	
	Male	0.83 (0.62 to 1.12)			
Education	≤ Elementary	1 (Reference)	0.219	—	
	>Elementary	0.76 (0.49 to 1.18)			
Religion	Muslim or other	1 (Reference)	0.206	—	
	Buddhist	0.73 (0.45 to 1.19)			
Employment status	Retired	1 (Reference)	0.134	1 (Reference)	0.106
	Unemployed	1.48 (0.89 to 2.48)	0.221	1.62 (0.98 to 1.02)	0.252
	Not in labor force	0.77 (0.50 to 1.18)	0.010	0.75 (0.47 to 1.22)	0.342
	Employed	0.62 (0.43 to 0.89)		0.80 (0.51 to 1.27)	
Income quartile	Low	1 (Reference)	0.566	—	
	Lower middle	1.13 (0.75 to 1.70)	0.457		
	Upper middle	1.17 (0.78 to 1.75)	0.266		
	High	0.78 (0.50 to 1.21)			
Alcohol use	Never	1 (Reference)	0.514	1 (Reference)	0.896
	Past	0.82 (0.45 to 1.49)	0.004	0.96 (0.50 to 1.83)	0.049
	Current	0.39 (0.20 to 0.74)		0.50 (0.25 to 0.99)	
Smoking tobacco	Never	1 (Reference)	0.268	—	
	Past	0.70 (0.38 to 1.31)	0.205		
	Current	0.72 (0.44 to 1.19)			
Physical activity	None	1 (Reference)	0.232	—	
	1–149 min/week ≥150 min/week	0.81 (0.56 to 1.15) 0.66 (0.42 to 1.04)	0.073		
Body mass index	Normal	1 (Reference)	0.156	1 (Reference)	0.584
	Under	1.46 (0.87 to 2.46)	0.728	1.17 (0.66 to 2.08)	0.946
	Overweight	1.09 (0.68 to 1.73)	0.006	1.02 (0.62 to 1.68)	0.007
	Obesity I	1.74 (1.18 to 2.58)	0.013	1.78 (1.17 to 3.61)	0.020
	Obesity II	2.03 (1.16 to 3.51)		2.01(1.12 to 3.61)	
Probable depression	No	1 (Reference)	0.032	1 (Reference)	0.726
	Yes	1.57 (1.04 to 2.35)		1.09 (0.67 to 1.78)	
Mental health	Good (80-100)	1 (Reference)	< 0.001	1 (Reference)	0.004
	Poor (< 80)	1.79 (1.32 to 2.43)		1.69 (1.19 to 2.41)	
Sleep problem	Rarely/sometimes	1 (Reference)	0.008	1 (Reference)	0.245
	Often/Mostly	1.62 (1.14 to 2.32)		1.29 (0.84 to 2.00)	
Hypertension	No	1 (Reference)	0.004	1 (Reference)	0.097
	Yes	1.54 (1.15 to 2.07)		1.34 (0.95 to 1.88)	
Diabetes	No	1 (Reference)	0.098	—	
	Yes	1.37 (0.94 to 2.00)			
Cardiovascular disease	No	1 (Reference)	0.014	1 (Reference)	0.722
	Yes	1.92 (1.14 to 3.25)		1.12 (0.59 to 2.13)	
Kidney diseases	No	1 (Reference)	0.045	1 (Reference)	0.129
	Yes	2.27 (1.02 to 5.05)		1.92 (0.83 to 4.47)	
Brain diseases and psychiatric problems	No	1 (Reference)	0.117	—	
	Yes	2.32 (0.81 to 6.64)			
Lung diseases	No	1 (Reference)	0.368	—	
	Yes	1.73 (0.52 to 5.71)			
Prescription drug	No	1 (Reference)	0.017	1 (Reference)	0.144
	Yes	1.53 (1.08 to 2.16)		1.33 (0.91 to 1.95)	
Functional disability	No	1 (Reference)	0.002	1 (Reference)	0.047
	Yes	2.55 (1.40 to 4.64)		2.04 (1.01 to 4.13)	

COR, Crude Odds Ratio; AOR, Adjusted Odds Ratio; CI, Confidence Interval.

## Discussion

The study found that the cross-sectional prevalence of SRA (4.0%) was lower than in previous studies among middle-aged and older adults in India (14.7%) (6), in six lower resourced countries (19.9% among women and 14.1% among men) (4), in China (31.4%) (7), in South Africa (24.7%) (5), and in Mexico (18). The lower prevalence of SRA in Thailand may be attributed to a lower rate of some risk factors, such as obesity, compared to other middle-income countries (7). Some other explanation for these country differences could be differences in the measurement of SRA, however, in all the studies cited here in India (6), China (7), Mexico (18), South Africa (5), and the six-country study (4) used exactly the same SRA, as in this study. However, the relatively low rate of SRA in Thailand appears to be confirmed in a community study in rural Thailand (≥15 years) with a prevalence of 11.3% osteoarthritis (based on radiographic and serological examinations) (39), and the global observation age-standardized incidence rates were the lowest in Southeast Asia (6.2), and the highest in high-income North America (22.5), South Asia (20.7), and Western Europe (20.4) (40).

We found that among women and those of older age, the prevalence of SRA was higher than among men and younger participants; however, this was not significant, unlike some previous research (7–11, 23). Some research studies found an association between lower education (10, 12) and SRA, but we did not find a significant association. The employment and higher economic status were in the unadjusted analysis protective against SRA, while in a study among older adults in Ghana the higher wealth status was associated with arthritis (9). In unadjusted analysis, being a Muslim or other increased the odds of SRA, which is contrary to a finding from a study in Thailand that found that the prevalence of radiographic knee osteoarthritis was significantly higher in Buddhists than in Muslims (41). The authors (41) attribute these differences to religious practices (“Muslims pray since childhood by forcing the knees into deep flexion, stretching the soft tissue surrounding the knee and decrease stiffness and contact pressure of the articular cartilage”).

In line with previous findings (5, 8, 14, 18, 24, 25), this study found a cross-sectional and incident association between obesity and SRA. Obesity may 'exhibit a chronic subclinical inflammatory state' increasing the risk of rheumatoid arthritis (42). Some studies found an association between physical inactivity and arthritis (12, 14, 15, 23, 43), while this study did not find this association. The non-significant association between physical inactivity and SRA in this study may be related to how physical activity was measured, it only included exercise and no other physical activity. In analyzing incident SRA, we found that current alcohol use was protective against arthritis, which is consistent with a review (17). The protective effect of alcohol use against arthritis may be explained “*via* attenuation of the innate inflammatory response” (17).

Furthermore, the study found associations between poor mental health (neurological or psychiatric problems, sleep symptoms, poor mental health and in unadjusted analysis probable depression) and SRA, which is consistent with previous results (5, 10, 18–21, 26–28). The association between sleep problems and arthritis may be related to pain at night (19). Prothero et al. (19) showed that “psychological interventions resulted in small to moderate improvement in biopsychosocial outcomes for patients with rheumatoid arthritis in addition to those achieved by standard care.” Furthermore, the relationship between poor mental health, such as depression, and SRA may also be bidirectional (44).

Consistent with some previous research (7, 10, 11, 19, 22), this study found a positive association between functional disability, hypertension, and in unadjusted analysis diabetes, cardiovascular disease, kidney disease and arthritis. It is possible that in these various physical conditions there is an underlying connection through pain and associated inflammatory dysfunction (10). Some determinants, such as sleep problems, or hypertension (7, 10), may also be consequences. For example, in this study, hypertension and sleep problems were positively associated with SRA in cross-sectional analysis but not in incident analysis. Furthermore, we found that the prevalence of SRA was higher among those who used prescription drugs, which is consistent with a study in the USA (43).

Study limitations include self-report evaluation, including diagnosed arthritis or rheumatism by a health care provider. The self-reported outcome may be limited due to common-method variance bias, recall or social desirability bias. Since only SRA was assessed, we cannot distinguish between different types of arthritis. The high attrition rate is a limitation for the longitudinal data. The study cannot establish causality due to confounding and reverse causality. Some variables that may affect arthritis, such as diet, were not included in this study but hopefully in the future. Future research should include multiple waves of HART to establish trajectories of SRA.

## Conclusion

One in twenty middle-aged and older Thai adults had SRA. Factors associated with cross-sectional and/or incident SRA included obesity, mental problems, sleep problems, prescription drug use, and functional disability. This information may be taken into account in the prevention and management of arthritis in Thailand and provide hints for future research.

## Data availability statement

Publicly available datasets were analyzed in this study. This data can be found here: Gateway to Global Aging Data, Health, Aging, and Retirement in Thailand: https://g2aging.org/?section=study&amp;studyid=44.

## Ethics statement

The studies involving human participants were reviewed and approved by Ethics Committee in Human Research, National Institute of Development Administration dmECNIDA (ECNIDA 2020/00012). The patients/participants provided their written informed consent to participate in this study.

## Author contributions

SP and KP conceived and designed the research, performed statistical analysis, drafted the manuscript, and made critical revision of the manuscript for key intellectual content. All authors fulfill the criteria for authorship, read and approved the final version of the manuscript, have agreed to authorship, and order of authorship for this manuscript.
